# Hyperactive deoxy-PIEZO1 shapes the circulatory life cycle of irreversibly sickled cells

**DOI:** 10.1016/j.bpj.2025.02.005

**Published:** 2025-02-08

**Authors:** Virgilio L. Lew, Simon D. Rogers

**Affiliations:** 1 Physiological Laboratory, Department of Physiology, Development and Neuroscience, University of Cambridge, Cambridge, United Kingdom; 2 School of Computing Science, University of Glasgow, Glasgow, United Kingdom

## Abstract

Sickle cell disease (SCD), affecting millions worldwide, is caused by the homozygous inheritance of the abnormal hemoglobin HbS. Deoxygenation of HbS in the venous circulation permeabilizes sickle cells to calcium via PIEZO1 channels, triggering a dehydration cascade driven by the outward electrochemical potassium gradient. This mechanism operates with particular intensity in a subpopulation of sickle red blood cells (RBCs), the irreversibly sickled cells (ISCs). The lifespan of ISCs is extremely short, about 4–7 days. Most of this time is spent in a profoundly dehydrated condition, the irreversibly sickled state, eliciting vaso-occlusion, which is considered the root cause of organ failure and pain crisis in SCD. There is a large experimental and clinical database on sickle cells and ISCs, but how ISCs form and evolve in the circulation remains a mystery. The present study is the first attempt to unravel the experimentally inaccessible life cycle of ISCs in vivo by applying a well-accredited model of RBC homeostasis and circulatory dynamics, using a vast array of validated experimental observations to tightly constrain the model parameters. The results showed that abnormally strong deoxy-PIEZO1 responses were needed for calcium to elicit a violent hyperdense collapse in ISC-destined stress reticulocytes within about a day in the circulation. The potassium-depleted ISCs remain in this maximally dehydrated but volume-stable condition, the pathogenic state, sustained by vigorous pump-leak balanced sodium fluxes. Eventually, sodium pump decay initiates rapid terminal rehydration by the unbalanced net gain of NaCl and water. Analysis of the mechanisms behind this three-stage circulatory life cycle of ISCs exposed a complex web of interactions among many components of the homeostatic fabric of RBCs. These findings point to the abnormally intense PIEZO1 response to deoxygenation in ISC-destined stress reticulocytes as a prime cause of ISC formation in vivo, a central target for future research.

## Significance

Irreversibly sickled cells (ISCs) are assumed to be the main cause of vaso-occlusion and organ failure in patients with sickle cell anemia. While much is known about ISCs experimentally, their life cycle in the circulation remains unknown. To bridge this experiment-circulation gap, we applied a well-tested model of red blood cell circulatory dynamics tightly constrained by experimentally verified facts. The results provided the first comprehensive account of the circulatory life cycle of ISCs and exposed abnormally powerful responses of PIEZO1 channels to deoxygenation as the prime cause of ISC formation and pathogenicity. This abnormal deoxy-PIEZO1 response emerges as a central target for future research and the search for new clinical and therapeutic interventions.

## Introduction

A large body of experimental results and clinical studies, accumulated over more than seven decades, indicates that irreversibly sickled cells (ISCs) play a critical role in the pathophysiology of sickle cell disease (SCD) (1,2,3,4,5,6,7,8,9,10,11,12,13,14,15,16,17,18,19,20,21). Three main samples of the varied nature of the evidence stand out. 1) Hydroxyurea, the current state-of-the-art treatment of SCD, selectively reduces the proportion of ISCs in the circulation of responding patients (22,23,24,25). 2) The fraction of ISCs is acutely reduced during pain crises, pointing to their selective trapping within the infarcted circulatory domains causing the crises (7,8). 3) In artificially perfused microcirculatory preparations, hyperdense ISCs were the dominant dense sickle cell fraction found in vaso-occluded areas (5,26).

There are four sickle cell subtypes in the circulation of patients with sickle cell anemia: reticulocytes, discocytes, ISCs, and F-cells, which contain a mix of fetal hemoglobin, HbF, and HbS. The proportions of these subtypes vary widely among patients. Clinical severity tends to be reduced in patients with high F-cell counts, particularly in those with higher proportions of HbF within the F-cells (27), suggesting that stimulating erythropoietic F-cell production would mitigate disease, which is currently under test in preliminary trials (27,28,29). On the other hand, no correlation was found between clinical severity and ISC counts (30,31), reflecting the need for an ISC presence but not high ISC counts in the complex processes leading to vaso-occlusion in different patients.

All sickle cell subtypes dehydrate in the circulation but at different rates (32). The dominant mechanism is always the same (13). Deoxygenation of HbS activates PIEZO1 channels, allowing Ca^2+^ influx down its steep inward electrochemical gradient. Elevated [Ca^2+^]_i_ activates the Ca^2+^-sensitive Gardos channels (KCNN4), eliciting net KCl and water loss. The circulatory lifespan of sickle cells is much reduced relative to the mean of 120 days for normal red blood cells (RBCs). The mean lifespan of F-cells is about 45 days, and that of discocytes is about 18 days (33,34).

As in many hemolytic anemias, sickle erythroid cells emerge from the bone marrow into the circulation as enucleated stress reticulocytes, a macrocytic immature reticulocyte containing residual mitochondria and endoplasmic reticulum and exhibiting elevated metabolism and membrane transport activity (35,36). Most stress reticulocytes expressing HbS mature more or less normally into sickle discocytes with 100% HbS and into F-cells (23,24).

However, the smaller population of ISC-destined stress reticulocytes embark on a markedly different path with a dramatically reduced 4–7 day circulatory lifespan (2). ISCs rapidly dehydrate (2), and their maturation and HbS biosynthesis are abruptly curtailed, rendering ISCs as immature cells with the lowest hemoglobin contents among sickle cell subtypes (12,14,37,38). ISCs remain in the arrested developmental condition of stress reticulocytes, with very high metabolic and membrane transport activities sustained almost to the end and with retention of residual organelles with ATP-dependent calcium-accumulating capacity (12,39).

Eaton (15) and Palek (40,41) were the first to document the elevated calcium contents of sickle cells, in the order of 40–100 *μ*mol/(10^13^ cells) in unfractionated sickle cell samples, over 10-fold the levels in normal RBCs. The elevated calcium of sickle cells was found within ISC vacuoles (12), indicating circulatory exposure to high intracellular calcium and a strong calcium accumulation capacity of the ISC vacuoles.

Notwithstanding the pathogenic relevance of ISCs, the dramatic volume transitions, and the mechanisms causing them during their brief circulatory life remain a mystery, a challenge this study attempts to resolve.

The model used in the present study is an extension of the ones recently applied to investigate the volume, ion transport, pH, and membrane potential changes human RBCs experience during oxy-deoxy transitions in the circulation in vivo (42,43,44). In order to investigate the circulatory life cycle of ISCs, the parameter values of the model were tightly constrained by a careful selection of verified experimental and clinical results on sickle cells, as explained under model constraints in methods.

The results emerging from the model simulations suggested that an abnormally strong PIEZO1 deoxy-response triggers an early ISC hyperdense collapse, a maximally dehydrated condition of potassium-permeabilized RBCs caused by accelerated depletion of the outward potassium gradient. The ISCs remain in this hyperdense but volume-stable and pathogenic state for most of the cells’ circulatory life cycle until late sodium pump decline leads to rapid terminal rehydration and lysis.

## Methods

### The RBC model

The RBC model version used for the present investigation (42,43,44,45,46) allowed exploration of days-long events with high time resolution and detail as needed to account for how very dynamic changes during brief inter-capillary transits shaped the days-long circulatory life cycle of ISCs.

Model simulations follow user-generated instructions recorded in editable protocol files (^∗^.txt). Protocols start by defining the constitutive properties of the RBC under study in an initial oxy steady-state condition, the reference state. Reference state entries are followed by sequences of dynamic state instructions designed to emulate physiological processes or experimental stages. The results used for the figures are reported in ^∗^.csv file format. The columns in the ^∗^.csv files display all the variables of the system. The rows report their changing values with time.

**Figure 1:**
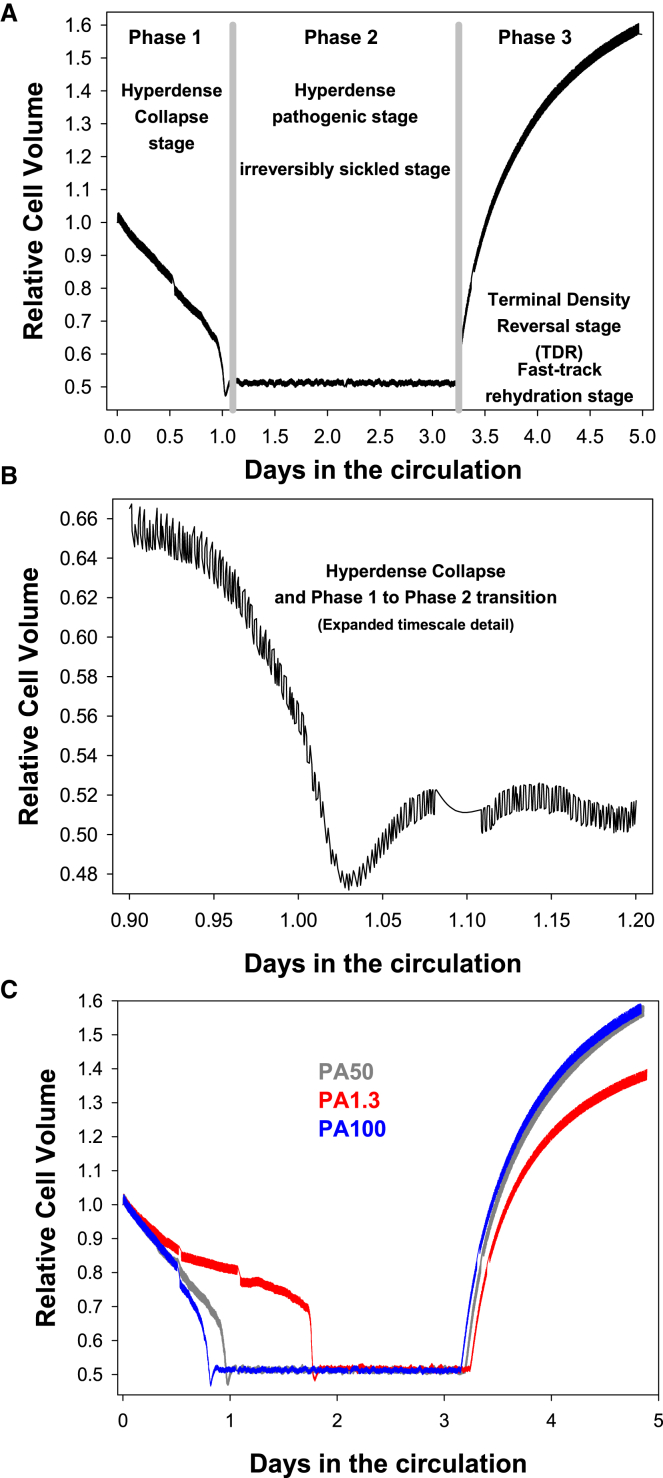
Predicted relative volume changes of a typical 5 day ISC as a function of time in the circulation. Effects of the deoxy-PIEZO1-mediated anion permeability (PA). The steep volume transitions in (*A*) allow a clear demarcation of the three different phases in ISC lifespans: the fast-track hyperdense collapse of phase 1 after about 1 day in the circulation; the extended volume stability of the hyperdense, irreversibly sickled, pathogenic state of phase 2; and the rapid, terminal rehydration of phase 3, elicited by sodium pump decline in the simulations. The expanded volume-time segment shown in (*B*) focuses on the hyperdense collapse period and the phase 1-to-phase 2 transition, exposing more clearly the volume oscillations resulting from the random variations in deoxy-Ca^2+^ permeabilities and inter-capillary transit times. The pattern shown in (*A*) fully complies with the constrains reviewed in methods, ready for an in-depth analysis of the homeostatic mechanisms at work in each of the three ISC phases. (*C*) illustrates the rate-limiting effects of the PA though deoxy-PIEZO1 channels on the initial dehydration and final rehydration rates of ISCs. Baseline PA for normal RBCs (68) = 1.3/h (*red*). Default deoxy-PA = 50/h (*gray*) (44). Non-rate-limiting PA = 100/h (*blue*).

**Figure 2:**
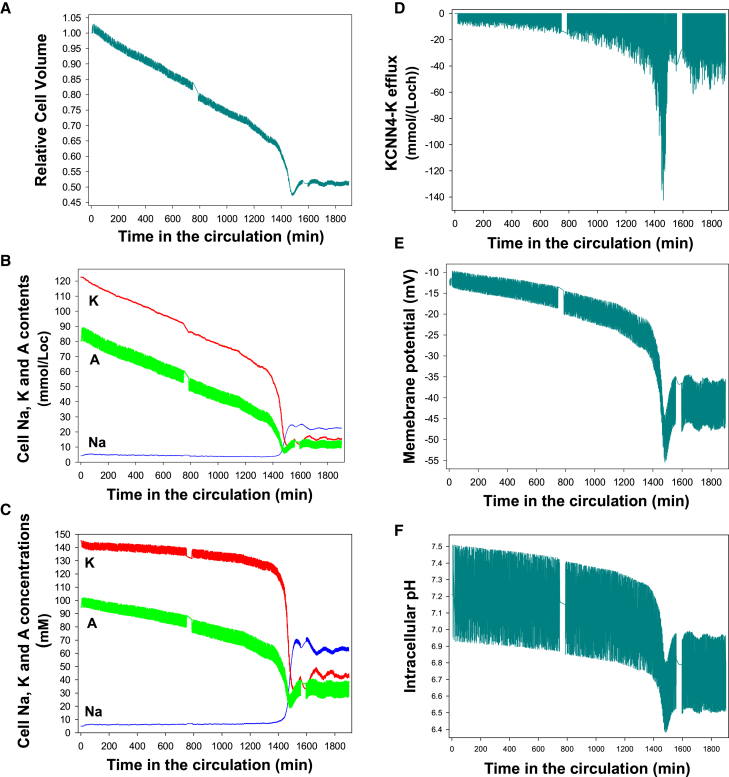
Predicted changes in selected homeostatic variables triggered by deoxy-PIEZO1 channel activation on ISC-fated stress reticulocytes entering the systemic circulation. Variable changes reported for the first 1.2 days in the life cycle of a 5 day typical ISC spanning phase 1 and the phase 1-to-phase 2 transition. Note the substantial variations in the amplitudes of the oscillations with which different variables respond to the random processes operating in the circulation. (*A*) Relative cell volume change showing a slow decline toward an accelerating steep trough before settling into a stable, profoundly dehydrated phase 2 volume level. (*B*) Changes in cell contents of Na, K, and diffusible anions (A = Cl^−^ + HCO_3_^−^). (*C*) Changes in the cell concentrations of Na, K, and A. (*D*) Gardos channel-mediated K efflux. (*E*) Membrane potential (Em) showing accelerating phase 1 hyperpolarization, settling to a stable hyperpolarized level upon entering phase 2. (*F*) Cell pH showing accelerating phase 1 acidification, settling to a stable mean acid pH in phase 2.

**Figure 3:**
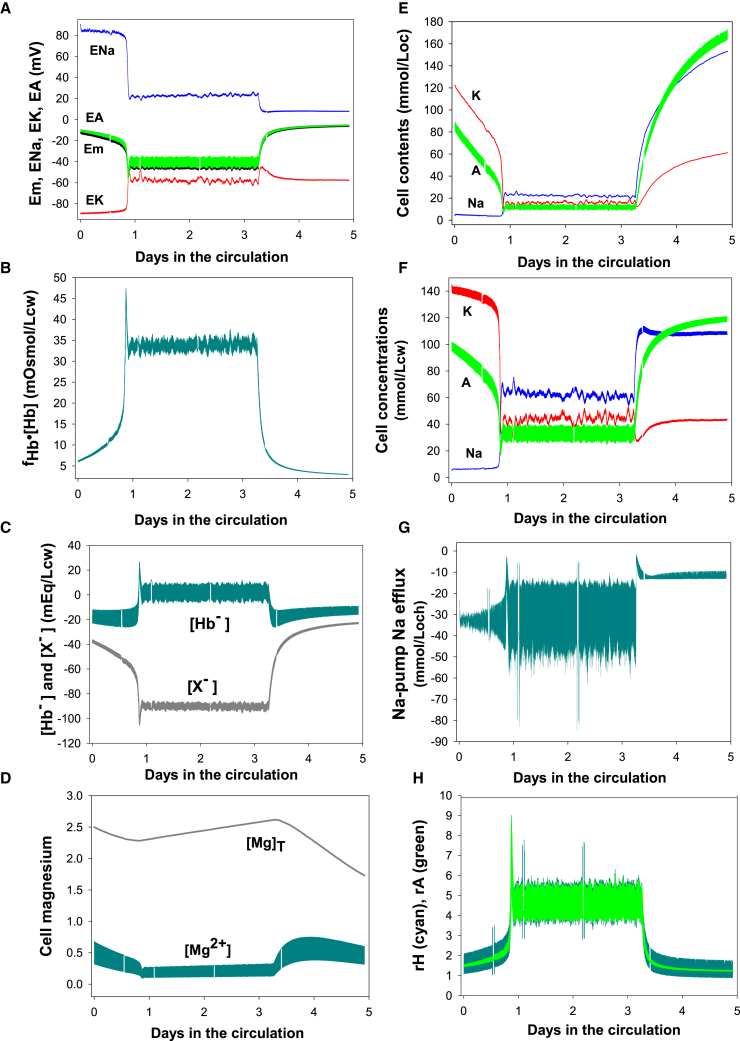
Predicted changes in selected homeostatic variables over the full 5 day lifespan of the typical ISC. Changes during phases 1 and 2 are triggered by PIEZO1 activation in the venous circulation. The transition from phase 2 to phase 3 was simulated by sudden sodium pump inhibition. (*A*) Comparisons between the cell membrane potential (Em) and the Nernst equilibrium potentials of Na (ENa), K (EK), and diffusible anions (EA). (*B*) Contribution of hemoglobin to cell osmolarity (mOsmol/Lcw) estimated from the product of the osmotic coefficient of hemoglobin, fHb (Osmol/mol), and the hemoglobin concentration CHb (mol/Lcw). (*C*) Concentration of charge (mEq/Lcw) on hemoglobin [Hb^−^ ] and X [X^−^ ], the impermeant organic and inorganic phosphate pools of the cell. (*D*) Total ([Mg]_T_) and free ([Mg^2+^]_i_) cell magnesium concentrations. The large difference is due to ATP and 2,3-BPG, the main cytoplasmic Mg buffers keeping most of the cell Mg bound (54,55,75). The oxy-deoxy oscillations in free [Mg^2+^]_i_ result from deoxy-hemoglobin binding of ATP and 2,3-BPG, thus releasing some [Mg^2+^]_i_ during deoxy transits. The increased Mg^2+^ permeability through deoxy-PIEZO1 mediates the changes in [Mg]_T_ driven by the electrodiffusional Mg^2+^ gradient across the plasma membrane (76), outwards during phases 1 and 3 and inwards during the hyperpolarized phase 2 (Figs. 2*E* and 3*A*). (*E* and *F*) Changes in cell contents and concentrations of Na, K, and A. (*G*) Sodium-pump-mediated Na efflux, oscillating up during oxy transits and down during deoxy transits. (*H*) Concentration ratios of diffusible anions (rA = CAo/CAi; *cyan*) and protons (rH = [H^+^]_i_/[H^+^]_o_; *dark green*) across the plasma membrane.

The updated RCM model version used here, RCM 1.0.2.jar, is available for download with open access from a GitHub repository (https://github.com/sdrogers/redcellmodeljava), together with the model code, comprehensive user guides, tutorials, an updated file with the governing equations of the model, and the protocol file of the “typical” 5 day ISC used for Figs. 1, 2, and 3, ready to be uploaded and run on RCM 1.0.2. The model operates as a program within the JAVA environment. RCM 1.0.2 was extended here to explore the effects of random variations within defined limits in the value of selected parameters (see model constraints). Ion permeabilities are reported in units of h^−1^ (by reference to the area/volume ratio of a standardized RBC), where 1 h^−1^ is equivalent to ∼2 × 10^−8^ cm/s. Ion fluxes are reported in mol (or subunits) per liter of original cells per hour (mol/Loch) standardized to an original volume of 10^13^ cells with a mean volume of ∼100 fL/cell. Concentrations per liter cell water are short-handed to /Lcw. A^−^ combines the two diffusible anions in the system, A^−^ = Cl^−^ + HCO_3_^−^. A and KA were used to represent A^−^, KCl, and KHCO_3_ in contexts where separate anion contributions to ongoing processes could not be mechanistically discerned (47).

### Simulation of oxy-deoxy-reoxy transitions

This was explained and reported in detail in the previous investigation of the effects of oxy-deoxy transitions on the circulatory dynamics of normal human RBC homeostasis (44). Briefly, the physiological connection between oxy-deoxy transitions and RBC homeostasis is the isoelectric point of hemoglobin, pI, which responds almost instantly to changes in pO_2_. To translate pI changes induced by oxy-deoxy transitions into cell pH changes, as required by charge conservation, we use the Dalmark equation (48,49,50), nHb = ɑ(pI – pH), where nHb is the net charge on hemoglobin in units of Eq/mol, and ɑ is the slope of the proton titration curve of Hb in intact RBCs (ɑ = −8 Eq/(mol^∗^Δ(pH – pI)) for HbS) (44,51,52). The pH changes, in turn, activate a reversible cascade of downstream homeostatic changes responsible for the well-known arterial-venous differences in RBC volume, pH, [Mg^2+^]_i_, and membrane potential (44,53,54,55). The model equations implementing the pI-pH translation were validated experimentally to within a 5%–7% coefficient of variation (52).

### Model constraints

In order to build a proper model representation of the homeostatic changes of ISCs throughout their short lifespan, it was necessary to categorize and select first the relevant experimental evidence to be rendered as model constraints. In this endeavor, it was essential to focus strictly on the reported results, often disregarding original interpretations lacking the benefits of later-gained hindsight knowledge. The constraining assumptions resulting from this exercise are reviewed next.

The HbS within ISCs was assumed to remain fully oxygenated or deoxygenated while traversing arterial or venular streams, respectively. ISCs dehydrate from stress reticulocyte to the hyperdense stage within around a day after bone marrow egress (2). PIEZO1 was assumed to be the main significant permeability pathway activated by deoxygenation of HbS, representing Psickle, the historical name under which most of the relevant experimental evidence on deoxy-induced ion permeabilization of sickle cells had been reported in the past (11,13,37,56). Deoxygenation of HbS was assumed to be necessary and sufficient to open PIEZO1 channels in sickle cells, regardless of the HbS concentration or its soluble, nucleated, or polymerized condition within the cell. Deoxy-PIEZO1 activation was assumed to instantly increase the electrodiffusional permeability of the plasma membrane to Ca^2+^, Mg^2+^, Na^+^, K^+^, Cl^−^, and HCO_3_^−^ with poor selectivity (57,58,59). In ISCs, the magnitude of deoxy-PIEZO1 permeabilization found necessary to induce hyperdense collapse within a day or so after bone marrow egress was assumed to persist throughout the full lifespan of the cells (14,37,38).

The deoxy-induced permeabilization of sickle cells was shown to vary stochastically over successive oxy-deoxy transitions (11), implying that the fraction of PIEZO1 channels that open in each cell during each deoxy episode varied at random within relatively wide limits. This stochasticity was represented in the model as a random variation in the amplitude of the PIEZO1 permeability in successive deoxy venous transits. A random variation within set time limits was also applied to the duration of oxy or deoxy inter-capillary transits, reflecting the variable duration of such transits in the systemic circulation.

Besides activating PIEZO1 channels, deoxygenation of HbS was assumed to also instantly block the spontaneous inactivation process, keeping the channel open for as long as HbS remained deoxygenated (56,60). Spontaneous inactivation functionality was assumed to be instantly restored on reoxygenation of HbS. The reversible block of spontaneous inactivation was also observed under sustained deformation of normal RBCs (58,61).

The initial reference state transport rates for the sodium and calcium pumps were set to values estimated by Wiley and Shaller (36) from measurements of tracer Na and Ca leaks and ouabain binding in reticulocytes about 10- and 40-fold higher, respectively, than in mature, normal, or sickle RBCs. The competence of ISCs to sustain the high metabolic rates of ATP supply required for such high pump fluxes was implicit in the model simulations (14,38,62).

The terminal density reversal rehydration of normal and sickle discocytes and F-cells was shown to be associated with a late decline in sodium pump activity (33,42,63). In ISCs, on the other hand, pump-mediated fluxes measured during the terminal fast-track rehydration stage (Fig. 1, phase 3) were about sevenfold higher than in normal RBCs or mature sickle cells (14,38), results that are apparently incompatible with sodium pump decline driving rehydration, a conflict resolved by the current investigation.

## Results

### Preliminary studies

#### Calcium pump-leak balance

A preliminary investigation was necessary to establish the feasibility of reconstructing the suggested pattern of ISC volume changes within the tight constraints set by the experimental observations listed under model constraints in methods. The modeling approach followed the minimalist perspective applied before (46) of proportioning the number of essential model components to the questions we may be able to answer within the availability of solidly verified facts.

Multiple preliminary simulations converged to show that for a hyperdense collapse to materialize within a day or so in the circulation (2), it was necessary to set the mean Ca^2+^ permeability of deoxy-PIEZO1 channels at levels about 10-fold higher than the magnitude of the flux attributed to the plasma membrane calcium pump in the initial reference state (in RCM units (43,44)). This elevated ratio sets the pump-leak dynamics of [Ca^2+^]_i_ changes during deoxy transits in the venous circulation.

#### The anion permeability of deoxy-PIEZO1 channels

A second important condition was that the electrodiffusional anion permeability attributed to deoxy-PIEZO1 channels, PA, should be substantially increased above the already high native anion permeability of the RBC plasma membrane. This proved necessary in order to reduce rate-limiting effects on the initial dehydration and final rehydration rates (Fig. 1 *C*).

#### Choice of a “typical” dehydration pattern to represent hyperdense collapse

Because of the random variations imposed on the duration of the inter-capillary transits and the Ca^2+^ permeability of deoxy-PIEZO1 channels, identical protocols never deliver identical results in successive simulations. However, the uniqueness of the overall pattern proved exceptionally robust. This enabled the investigation of mechanisms to be centered on a “typical” representative pattern, a choice shown in Fig. 1 *A* for a 5 day ISC with the pump-leak turnover rates for the calcium and sodium pumps set to default initial values of 36 and 26 mmol/Loch, respectively, values that deliver initial pump fluxes within the range measured by Wiley and Shaller (36). In methods (the RBC model), it is explained how to access the RCM model, upload the protocol of the typical 5 day ISC, and run the program from which the information shown in Figs. 1, 2, and 3 is derived.

#### Testing effects of cell lingering in the venous circulation

The pattern of Fig. 1 was not altered by interspersing in the simulations episodes of ISCs trapped in venules to represent lingering events suggested to influence ISC dehydration rates (1,2). Effects of 40 min lingering episodes in deoxy conditions on different homeostatic variables can be seen in Fig. 1 *A* at 0.5 and 3.5 days, in Fig. 1 *B* at about 1.1 day, and in Figs. 2 and 3 as brief disruptions in ongoing patterns, with hardly a noticeable dent in the overall trend of variable changes. The sturdy invariance of the central pattern suggests no major role of lingering episodes on the overall homeostatic responses of ISCs.

Relative to the power required for the deoxy-PIEZO1-calcium dehydration cascade to trigger rapid hyperdense collapse in ISCs, the contribution of the K:Cl cotransporter (64,65), represented in the model as described before (46), was hardly detectable. Inclusion of K:Cl in the current study was therefore not considered of value. If these results are verified, they would invalidate the early suggestions (13,37) that K:Cl cotransporters contribute significantly to ISC dehydration. On the other hand, the tests confirmed the K:Cl potential to add substantially to the densification profiles of slower dehydrating sickle cells, discocytes and F-cells, which dehydrate over periods of weeks (66,67).

### Mechanisms driving the changing homeostasis of ISCs

#### Phase 1: The hyperdense collapse

Circulatory hyperdense collapse is an ingrained liability of human RBCs resulting from the complement of ion transporters in the plasma membrane (42). For normal RBCs, sickle discocytes, and F-cells, reaching the tipping point is a slow process, and the collapse is prevented by late decline in Na pump activity causing terminal density reversal (38,63). This decline allows aging cells to regain NaCl and water and thus reverse the progressive densification trend and extend the rheological competence and lifespan of the cells (69).

For ISC-destined stress reticulocytes, on the other hand, circulatory emergence initiates an unstoppable fast-track dehydration process toward a hyperdense tipping point (Figs. 1 and 2 *A*), the result of feedback loops entangling a web of dynamic interactions among many components of the homeostatic fabric of the cell, a web we attempt to disentangle next, step by step.

Each deoxy transit in the venous circulation activates a random fraction of PIEZO1 channels (11) that remain open for the duration of the transit, allowing Ca^2+^ influx down its steep electrochemical gradient, elevating [Ca^2+^]_i_. Elevated [Ca^2+^]_i_, in turn, activates Gardos channels (Fig. 2 *D*), causing net loss of KA (Figs. 2 *B* and 3 *E*) and water (Figs. 1, *A* and *B*, and 2 *A*), driven by the outward electrochemical potassium gradient.

During oxy transits, with oxy-PIEZO1 channels closed, [Ca^2+^]_i_ levels are rapidly restored to baseline resting levels by the plasma membrane calcium pump (PMCA), and Gardos channels fall silent to a zero net K-flux baseline (Fig. 2 *D*). Over many oxy-deoxy transits, progressive hyperpolarization (Figs. 2 *E* and 3 *A*) gradually increases the driving force for Ca^2+^ influx through deoxy-PIEZO1 channels, accelerating KA losses (Figs. 2 *B* and 3 *E*) and dehydration (Figs. 1 and 2 *A*). In addition, within each sequential deoxy transit, the potential-enhanced Ca^2+^ influx generates progressively higher time-integrated [Ca^2+^]_i_ exposures of the Gardos channels (Fig. 2 *D*), contributing to accelerate dehydration toward a volume trough (Figs. 1, *A* and *B*, and 2 *A*), with extreme depletion of the cell’s potassium and diffusible anions (Figs. 2 *B* and 3 *E*). Hyperpolarization (Fig. 2 *E*) is the main accelerator of dehydration. It is, therefore, important to trace its origins.

The ground permeability of RBC plasma membranes to diffusible anions, Cl^−^ and HCO_3_^−^ (lumped under A in the model user interface), is at least two orders of magnitude higher than the ground permeability to Na^+^ or K^+^ (70,71). Therefore, the membrane potential of RBCs, Em, follows closely the Nernst equilibrium potential of the diffusible anions, EA, with Em ∼ EA (Fig. 3 *A*). EA, in turn, is determined by the plasma/cell concentration ratio of the diffusible anions (rA), rA = CAo/CAi = ([Cl^−^]_o_ + [HCO_3_^−^]_o_)/([Cl^−^]_i_ + [HCO_3_^−^]_i_). At constant plasma concentration levels of Cl^−^ + HCO_3_^−^, the ISC anion concentration ratio increases sharply as CA falls during phase 1 (Figs. 2 *C* and 3 *F*), gradually hyperpolarizing the membrane (Figs. 2 *E* and 3 *A*). Throughout the following text, CA, CK, and CNa will be used as shorthand for the intracellular concentrations of Cl^−^ + HCO_3_^−^, K^+^, and Na^+^, respectively.

With increasing hyperpolarization resulting from the steep fall in diffusible anion concentration (A), the next question is what causes CA to fall so much faster than CK, creating an apparent electroneutrality breach (Figs. 2 *C* and 3 *F*). The short answer is that as the cells dehydrate, the increasing concentration of the negatively charged impermeant anions ([X^−^ ], Fig. 3 *C*) displaces diffusible anions to maintain electroneutrality and thus causes CA to fall faster than CK. But changes in CA also have a profound effect on cell pH.

At constant plasma CA concentrations, CAo, the fall in cell CA, CAi, increases the anion concentration ratio across the plasma membrane, rA = CAo/CAi (Fig. 3 *H*). RBC pH is controlled by the Jacobs-Stewart mechanism (47,72) operating via the anion exchanger (AE1) and the CO_2_ shunt to rapidly restore the equality of concentration ratios of protons, rH = [H^+^]_i_/[H^+^]_o_ and diffusible anions rA = CAo/CAi toward rH = rA (Fig. 3 *H*). At constant extracellular pHo and CAo, the fall in CAi leads to an increase in [H^+^]_i_. As shown in Fig. 2 *F*, the ISC cell pH gradually shifts from initial values around 7.2 toward a ∼6.4 dip before recovering to a mean around 6.7, with oxy-deoxy oscillations spanning the 6.6–6.9 range upon transition to phase 2. This change in intracellular pH strongly influences the net charge on hemoglobin ([Hb^−^ ], Fig. 3 *C*) from an initial value of about −20 mEq/Lcw to a slightly positive mean value during oxy-deoxy oscillations as the cell pH falls below the pI of HbS (set to 7.4 at 37°C in the model (51,73). Electroneutrality is maintained by compensatory changes in [X^−^ ] and CA.

As ISCs dehydrate, the power function increases in the osmotic coefficient of hemoglobin (74), fHb, causing the osmotic contribution of hemoglobin to increase from about 7 to 45 mOsmol/Loch at the trough of the hyperdense collapse (Fig. 3 *B*). The resulting increase in colloidosmotic pressure causes progressive water retention within the cells and an increasingly hypertonic KCl effluent upon Gardos channel activation during venular transits (47,68), with hypertonicity rapidly dissipated in the circulatory flow.

This analysis reveals the mechanisms driving the entangled web of interactions leading to the hyperdense collapse of ISCs during phase 1 and the transition to phase 2. For the speed with which this mechanism operates in ISCs, the decisive factor is the magnitude of the deoxy-PIEZO1-mediated increase in permeability, the first step in the chain of phase 1 interactions.

#### Phase 2: The hyperdense pathogenic stage

After the volume trough of the hyperdense collapse, the pathogenic phase 2 starts with the cells retaining their hyperdense, K-depleted condition (Fig. 1 *B*; see also Fig. 2, *A–F*, after day 1). The oxy-deoxy transitions in the circulation cause all variables to oscillate with amplitudes determined by the processes controlling the kinetic responses of each of the variables (Figs. 2 and 3). Against this dynamic rumbling background, the most important property of stage 2 is the remarkable overall stability of the cell volume and all other homeostatic variables (Fig. 3).

Phase 2 volume stability is the result of balanced Na fluxes between deoxy-PIEZO1-mediated inward Na leaks and sodium-pump-mediated Na extrusion in K-depleted cells (Figs. 2 *B* and 3 *E*). The amplitude variations in the oxy-deoxy oscillations of the sodium-pump-mediated sodium efflux in Fig. 3 *G* show a gradual increase during phase 1 toward a maximal amplitude and stable range in phase 2. This pattern reveals a progressive increase in sodium pump activity during phase 1 toward a very dynamic and stable pump-leak Na-flux balance during phase 2. How does the model account for the gradual increase in sodium pump activity toward the balancing phase 2 level?

The kinetics of the fall in the cell potassium concentration (Figs. 2 *C* and 3 *F*) provide the answer. The normal high intracellular K concentration exerts a powerful inhibitory effect on the affinity of the sodium pump for intracellular Na (77,78). As the intracellular K concentration falls, inhibition relaxes. The pump-mediated Na efflux (Fig. 3 *G*) increases progressively in response to the combined effects of the fall in the cell K concentration and the slowly increasing intracellular sodium concentration (Figs. 2 *C* and 3 *F*). Pump-leak balance is attained when the K-gradient is exhausted, and the pump attains its highest kinetic responsiveness to intracellular sodium concentration changes upon reaching phase 2.

Phase 2 stability masks a highly dynamic condition of pump-leak turnover and metabolic activity (35,79). Although Ca^2+^ influx through deoxy-PIEZO1 channels cannot dehydrate the K-depleted cells any further, elevated [Ca^2+^]_i_ continues to activate the PMCA and all ATP-dependent calcium accumulating processes throughout all phases of the ISC lifespan. Even neglecting ATP consumption by vacuolar calcium accumulation and other processes, sustaining transport-related ATP turnover by the plasma membrane pumps on their own requires already a rate of glycolytic ATP production 20-fold above that in mature, normal RBCs or sickle discocytes. Only the persistence of an immature stress reticulocyte condition in ISCs can explain the maintenance of such a powerful level of metabolic and membrane transport activity.

#### Phase 3: Fast-track terminal rehydration

A brief detour is needed here to explain the nature of the experimental data on which our knowledge of the condition of phase 3 ISCs is based. Cells recovered from the lowest-density fractions after sickle cell density fractionations contain a mix of sickle cells in different stages of their lifespans: 1) reticulocytes and stress reticulocytes, 2) young discocytes, 3) young F-cells, 4) ISC-fated phase 1 stress reticulocytes, and 5) reticulum-free, phase 3 rehydrating ISCs, the only potassium-depleted cells in that density fraction. This made it possible to separate phase 3 from all the other light-density, high-K cells by suspending the mix in calcium-containing, low-K media and permeabilizing them to K using a calcium ionophore or valinomycin. The K-depleted, phase 3 ISCs retain their original density resisting calcium- or valinomycin-induced dehydration. All others will dehydrate and densify by KCl and water loss, allowing further density fractionation to separate enriched phase 3 ISCs. Experimental studies on these ISCs confirmed their low HbS contents, inverted Na-K gradients, and puzzlingly high levels of Na pump-mediated fluxes (14,38).

Paradoxically, it is this high level of Na pump activity in phase 3 that holds the only experimentally based clue we have to suggest that Na pump inhibition is the mechanism triggering ISC rehydration upon transit from phase 2 to phase 3 (Fig. 1 *A*). Pump-mediated Na fluxes oscillate between −50 and −15 mmol/Loch during phase 2 (Fig. 3 *G*). After transition to phase 3, model predicted Na fluxes settled around −10 mmol/Loch (Fig. 3 *G*), close to measured phase 3 fluxes around −7 to −9 mmol/Loch (14,38). Such values appear unaccountably high when compared with those in mature normal or sickle RBCs, but they actually represent a substantial level of terminal Na pump inhibition relative to phase 2 levels. In this light, sodium pump decline appears as the main or only trigger required to explain the phase 2 to phase 3 transition.

We have no clues for when or why sodium pump decline would start along phase 2 or what its true inhibition kinetics may be. On this level of ignorance, the simplest approach was to simulate a single sudden inhibitory step at an arbitrary time along phase 2. Similar results were also generated by playing with gradual inhibitory steps, a way of representing progressive metabolic decline, a reasonable speculation after such an intense phase 2 metabolic period but so far only an idea awaiting experimental scrutiny. The activity decline of membrane transporters in normal RBCs, as documented for Gardos channels (80) and the calcium (81,82) and sodium (33,42) pumps, differs substantially in pattern and timing along the cells’ lifespan. The calcium pump decline follows an exponential pattern from reticulocyte onwards, whereas sodium pump decline starts after about 80–100 days (42,83).

The results shown in Fig. 3 *D* tested the hypothesis that gradual deoxy-induced Mg depletion of the ISCs during phase 2 could have reduced [Mg^2+^]_i_ sufficiently below half-maximal activation of the sodium pump by [Mg^2+^]_i_, measured at about 50 *μ*M (84), to cause sufficient pump inhibition and thus initiate rehydration. The results proved interesting but negative: negative because [Mg^2+^]_i_ could never be reduced enough to inhibit the pump in circulatory conditions, and interesting because the increase in total Mg during phase 2 was surprising. It was ultimately explained by the hyperpolarized membrane potential condition during phase 2 (Fig. 3 *A*) reversing the [Mg^2+^] gradient in the inward direction, thus preventing further reduction in [Mg^2+^]_i_ toward inhibitory levels for the pump.

From these considerations, the most likely explanation for the mechanism of ISC rehydration in phase 3 would seem to be that at some stage along phase 2, metabolic exhaustion limits the ATP supply to the pumps. The decline in Na pump activity allows progressive NaCl gains by cells no longer able to balance the large deoxy-PIEZO1-mediated inward NaCl leaks (Fig. 1 *C*).

## Discussion

The results in this study provide the first comprehensive account of the life cycle of ISCs in the circulation by integrating a vast episodic database on sickle cells into a sequential three-phase circulatory cycle. A detailed analysis of the model results exposed the driving forces and mechanisms behind the dynamic phase transitions (Figs. 1, 2, and 3). Here, we focus on some of the main open questions prompted by this study.

The results showed that in order to set stress reticulocytes on the ISC path along the pattern represented in Fig. 1 *A*, deoxy-PIEZO1 permeabilities ought to be set at least one order of magnitude above the maximal calcium extrusion capacity of the plasma membrane calcium pump (in RCM units) in each cell, a huge bespoke conductance level. This prediction, together with the additional findings in this study, open up a pandora box of questions, a challenging prompt for future research.

The three sickle cell subtypes with ∼100% HbS in the circulation of sickle cell anemia patients are discocytes, ISCs, and HbF-free stress reticulocytes. Hidalgo et al. (85) showed that erythropoietin receptor signaling increases the number and speed of erythroblast cell cycles and also the size of the emerging RBCs in experimental conditions. Could this be the production path of stress reticulocytes in vivo? Experimentally produced stress reticulocytes under fully controlled conditions could become a formidable cell source for the study of stress reticulocyte properties. We could then attempt to answer at least some of the critical questions emerging from the results of the present study.

What determines stress reticulocytes to mature to discocytes or follow the ISC path? Is the ISC path preset before bone marrow egress? Do HbS stress reticulocytes emerge with a wide distribution of PIEZO1 channel densities in their membranes, those at the upper end evolving to ISCs and those at the lower end maturing to discocytes? What causes the arrested developmental condition of ISCs? Could the violence of the hyperdense collapse block ISC maturation and terminate hemoglobin biosynthesis?

Why would PIEZO1 channels behave differently in ISC-destined stress reticulocytes? PIEZO1 kinetics are strongly influenced by the lipid composition of the membrane (86,87,88) and perhaps also by interactions with cortical cytoskeletal components. Both membrane lipids and cytoskeletal interactions are in very dynamic flux during cell maturation (35), suggesting that a subpopulation of stress reticulocytes may have unique membrane lipid and cytoskeletal component profiles influencing deoxy-PIEZO1 responses. Do these features remain at their immature stress reticulocyte condition throughout the ISC life cycle? How is the spontaneous inactivation process of PIEZO1 channels reversibly blocked by HbS deoxygenation? There are no answers yet to any of these questions.

HbS polymerization occurs as a very steep power of the deoxy-HbS concentration (16,17). Deoxy-HbS polymers deform the cells into the proverbial sickling shapes (4), activating stress-sensitive PIEZO1 channels and leading to sickle cell dehydration. ISCs challenge the simplest versions of this polymer-permeability connection. Deoxygenation of light, reticulocyte-rich sickle cell fractions with reduced HbS concentrations and hardly detectable sickling had more powerful permeabilizing and dehydrating effects than those measured in denser, massively sickled, discocyte-rich fractions (14,37). Could ISC-fated stress reticulocytes, with macrocytic, large-volume, low-HbS cells just emerging from the bone marrow, already house a nucleating polymerization grain when starting to deoxygenate in the circulation? Could they be emerging from deoxygenated niches in the bone marrow that already contain nucleation seeds?

Analysis of delay times preceding fiber formation (21,89) suggests that in vivo deoxy-HbS fiber formation is very far from equilibrium. Do these studies offer alternative mechanisms for deoxy-HbS activation of PIEZO1 channels in stress reticulocyte membranes? Deoxy permeabilization of reticulocyte-rich sickle cells was shown to be powerfully stimulated by heparin and inhibited at high bumetanide concentrations (14,37), a PIEZO1 response unique to ISCs. Could these effects be hinting at a deoxy-HbS mechanism of PIEZO1 channel activation that bypasses the polymer-permeability connection? Are the heparin and bumetanide effects mediated by direct or indirect interactions with PIEZO1?

The calcium accumulation process of ISCs generates additional questions. Electron probe x-ray microanalysis of ISCs (12,90) revealed vacuolar calcium accumulation in amounts high enough to fully account for the elevated mean total calcium content of sickle cells. The P/Ca ratios within the vacuoles varied from 1.5 (the ratio in hydroxyapatite) to 7.6 (12). Accumulation was also shown to be ATP dependent, suggesting PMCA or smooth endoplasmic reticulum pump participation. Altogether, the meagre evidence available on the calcium accumulating vacuoles is compatible with mitochondrial, endoplasmic, or endocytic origins. The identity of the vesicles, their relative proportions, whether or not they have any influence on the dynamics of the [Ca^2+^]_i_ changes determined by the deoxy-PIEZO1-PMCA pump-leak balance at the plasma-membrane level, and whether calcium accumulation occurs evenly or unevenly throughout the life cycle of the ISCs all remain open questions at present.

The results of this study offer a new perspective on the circulatory life cycle of ISCs fully consistent with a large body of experimental and clinical evidence. The predicted three-stage life cycle offers a unifying perspective on many seemingly unconnected experimental results. We hope that the predictions advanced in Figs. 1, 2, and 3 will stimulate experimental tests of their validity and that the wealth of questions opened by this new perspective may eventually lead to therapies able to prevent the abnormal intensity of the deoxy-PIEZO1 response in ISCs.

## Acknowledgments

We are grateful to Daniel J. Lew, Teresa Tiffert, and Pietro Cicuta for helpful comments on early versions of the manuscript. This work was supported by funds from the 10.13039/501100000735University of Cambridge and the 10.13039/501100000853University of Glasgow.

## Author contributions

The biophysical, medical, and computational components of this research were discussed and drafted in close collaboration by both authors.

## Declaration of interests

The authors declare no competing interests.
